# Assessment of Knowledge and Attitudes Regarding Acute Pediatric Pain Management Among Anesthesiologists, Pediatricians, and Pediatric Nurses: A Cross-Sectional Study from Jordan

**DOI:** 10.3390/healthcare13202570

**Published:** 2025-10-13

**Authors:** Anas Alrusan, Rania Al-Bataieneh, Ala”a Alhowary, Saif Aldin Rawabdeh, Mohammad Al Hazaymeh, Mohammad Elhammdan, Ali Al-Ali, Sara Alhaj Omer, Obada Matalkeh, Shahed Shloul, Lana E. Obeidat, Lubna N. Bataineh, Diab Bani Hani

**Affiliations:** 1 Department of Anesthesiology, Faculty of Medicine, Jordan University of Science and Technology, Irbid 21110, Jordan; 2 Faculty of Medicine, Jordan University of Science and Technology, Irbid 22110, Jordan; 3 Department of Pediatrics, Faculty of Medicine, Jordan University of Science and Technology, Irbid 22110, Jordan

**Keywords:** pain management, pediatric, knowledge and attitude survey regarding pain, healthcare, anesthesiologists

## Abstract

**Background:** Pain is one of the most common complaints among all age groups. Adult patients can express pain more clearly. Unfortunately, pediatric patients cannot perform this. This study aimed to assess the extent of knowledge of healthcare providers regarding acute pediatric pain management. **Methods**: A cross-sectional study was conducted utilizing the modified Knowledge and Attitude Survey Regarding Pain (KASRP) questionnaire through face-to-face interviews. The modified version included 34 questions (24 true/false questions and 10 MCQs). This study targeted anesthesiologists, pediatricians, and pediatric nurses through interviews. Demographic and educational data were analyzed as factors affecting the results of the KASRP questionnaire. The total score was classified as poor, fair, or good. **Results**: A total of 137 participants were enrolled in this study. The mean age was 31.8 years, and of the participants, 62.8% were women, 30.0% were anesthesia physicians, 25.5% were pediatric physicians, and 44.5% were pediatric nurses. The participants scored an average of 20.7 out of 34. Performance was categorized as poor, fair, or good, with 22.6%, 64.2% and 13.2% of participants falling into each category, respectively. The mean score of correct responses was higher for anesthesiologists (*p* = 0.0001). Specialists achieved higher mean scores than residents. Completion of pediatric pain management courses and the use of assessment tools were linked to higher performance. **Conclusions**: Pediatric nurses achieved lower scores for knowledge of acute pediatric pain management than physicians. Anesthesiologists achieved the highest score, probably because of their training in pain management. All healthcare providers should attend pain management courses.

## 1. Introduction

Pain is the most common presenting symptom worldwide and is a sensory, physical, emotional, and psychological experience. The individualized nature of pain makes its assessment a complex process, as pain occurs across a spectrum of conditions, including acute injuries, recurrent or chronic pain, and pain related to chronic diseases. Acute pain differs from chronic pain. While acute pain is typically temporary, eliminated with the healing of an injury [1], chronic pain, on the other hand, may or may not be related to an underlying, ongoing disease. Chronic pain can persist long after an initial injury has healed (longer than 3 months) [2]. Accordingly, the process of pain evaluation is complex and can be affected by personal attitudes and the various forms in which pain can exist. Certainly, pain is one of the most important concerns have medical staff for their patients. Therefore, the extent of pain management awareness in all age groups, especially in pediatric age groups, must be excellent. In recent years, several studies have been conducted and concluded that the assessment of pain by healthcare providers remains poor in clinical practice among all age groups [3,4]. The most widely accepted definition of pain has been developed and refined over time by the International Association for the Study of Pain (IASP), which defines it as “an unpleasant sensory and emotional experience associated with, or resembling that associated with, actual or potential tissue damage” [5,6]. Pain can be classified as acute or chronic based on its duration. Acute pain typically has a sudden onset and is short-lived, while chronic pain persists beyond the expected period of tissue healing [2]. While adults are generally able to understand and articulate their feelings and experiences, children, particularly infants and toddlers, often cannot. Therefore, accurately assessing pediatric pain requires careful observation of subtle physiological and behavioral changes associated with this unpleasant sensation [7]. Observable changes may include crying, motor responses, and alterations in facial expression, posture, appetite, activity level, and general appearance. Additionally, variations in vital signs may also be present [8]. Early intervention in response to pain is essential for ensuring pediatric well-being. Unrecognized and undertreated pain can negatively impact a child’s development, increase psychological distress (such as fear and anxiety), and potentially progress into chronic pain that persists into adulthood [2,9].

In a study conducted by Peker et al., they suggested several steps to overcome some of the limitations of proper pain management [3]. Most healthcare providers realize that healthcare systems present more significant barriers to proper pain management than patients themselves. Furthermore, half of them reported that legal regulations regarding the prescription of opioids have some influence [3]. Another study by Kim et al. demonstrated that there was a significant difference in pain management scores between nurses and doctors, and pain education should be conducted for both [4].

A study conducted by Schafheutle et al. regarding possible reasons for suboptimal pain management among nurses recognized a number of barriers, such as workload and lack of staff, and also legal and institutional constraints [10]. Nurses further reported that analgesic prescription was sometimes insufficient, or that medication reviews were suboptimal [10]. Nurses’ attitudes and judgements regarding pain management were further highlighted in replies to a number of attitude statements and a question about the aim of administering analgesia [10]. A study in Jordan revealed that nurses in Jordan have a shortage of knowledge regarding pain management and a discrepancy between their judgments and actual practices. In addition, nurses in Jordan showed a lower level of pain knowledge than that reported worldwide [11]. This study aims to demonstrate the extent of knowledge of healthcare providers (anesthesiologists, pediatricians, and pediatric nurses) regarding acute pediatric pain management. Moreover, this study aims to investigate the differences in knowledge of acute pediatric pain management among different groups of healthcare providers and the factors influencing this knowledge.

## 2. Material and Methods

### 2.1. Patients and Study Design

After obtaining ethical approval from the Institutional Review Board at Jordan University of Science and Technology, a descriptive cross-sectional study was conducted to evaluate and measure the knowledge of pediatric-related healthcare providers at King Abdullah University Hospital regarding acute pain management in pediatrics. This study was performed utilizing a specialized questionnaire, the Knowledge and Attitude Survey Regarding Pain (KASRP), from September 2024 to May 2025. Date was collected through in-depth interviews. In addition to the questionnaire, the included data comprised demographic information (age, sex, and marital status). Information regarding education and experience was also obtained and included total years of experience, average working hours per week, and educational certificates (for pediatric nurses, bachelor’s or master’s degrees). Furthermore, the roles and fields for pediatric nurses were obtained and categorized into pediatric emergency department nurses, pediatric ward nurses, and inpatient pediatric intensive care unit nurses. Physicians were classified as pediatric or anesthesia physicians, and specialists or training residents. Moreover, data on pain management skills, such as whether they attended a formal or informal course on pain management, were gathered. Electronically signed informed consent was obtained from the participants. This study was conducted in accordance with the Declaration of Helsinki, good clinical practices, and relevant regulatory guidelines.

This study included all healthcare providers who deal with the pediatric age group. Pediatricians, anesthesiologists, and pediatric nurses (in emergency departments, inpatient wards, and intensive care units) were enrolled in this study. Newly hired nurses (less than two months), nursing students, and nurses from operation rooms, nursing administration, and nursing education departments were not invited to participate. Any participant who had no experience in dealing with patients in the pediatric age group was excluded.

The assessed outcomes of the study were the average mean score for the healthcare providers’ level of knowledge and attitudes towards acute pediatric pain management, and the factors affecting the levels of knowledge and attitudes towards acute pediatric pain management.

### 2.2. Study Settings and Data Collection

KAUH is the only tertiary academic hospital in northern Jordan and is the main referral center in the region that provides access to all healthcare services for the pediatric population. Consultations are available for all pediatric and surgical subspecialties, along with access to interdisciplinary pain services. All registered nurses work full-time in the pediatric clinical departments.

First, we obtained a list of all names of targeted healthcare providers from hospital records. Then, the healthcare providers were visited during their break time and invited to participate. Recruitment was conducted through face-to-face interviews by two well-trained research assistants. After explaining the aim and process of the study and obtaining approval from the participants, the research assistants offered the participants the KASRP questionnaire electronically on Google Forms. Each participant completed the questionnaire under the supervision of the research assistant.

### 2.3. Subjective Scale and Questionnaire

A well-validated self-reported questionnaire instrument was utilized in this study: the KASRP questionnaire [5]. The original KASRP questionnaire is composed of 22 true/false questions, 14 multiple-choice questions (MCQs), and 2 case scenarios, each followed by 2 MCQs related to the given scenarios. This resulted in a total of 40 responses. Regarding the psychometric properties of the KASRP tool, the content validity of the tool was established by the original authors, Ferrell and McCaffery [5]. Subsequent studies that used the KASRP questionnaire have reported internal consistency measures ranging between 0.76 and 0.82 [12,13]. Furthermore, KASRP has been validated on medical staff and nurses of various levels of expertise and has seen widespread application for assessing levels of knowledge and attitudes regarding pain treatment [14,15,16,17,18,19]. The questionnaire was designed to test knowledge of pain management and attitudes regarding characteristics of pain management, pharmacology, addiction, physical dependence, tolerance, and principles of assessment and management.

The KASRP questionnaire was modified in this study because the study focused on acute pain management, and due to cultural barriers in Jordan. Accordingly, for those two reasons, questions related to cancer pain management and drug abuse were omitted. Questions 9, 10, 16, and 20 from the true/false section, questions 23, 25, 28, 30, 32, and 33 from the MCQ section, and the two case scenarios were removed. To compensate for this, we added eight validated questions to the questionnaire (six true/false questions and two MCQs). Subsequently, the total number of questions in our modified version was 34 (24 true/false questions and 10 MCQs). The total and average scores of the participants were classified as poor performance (≤50%, or ≤17 correct responses), fair performance (>50% and ≤75%, or >17 and ≤25 correct responses), and good performance (>75%, or >26 correct responses). The total performance of the participants and the performance for each question were calculated.

As our staff and healthcare providers are trained in speaking English, and English is one of the requirements for employment, the questionnaire utilized in this study was in the English language. The specific modified KASRP questionnaire is provided in the Supplementary Materials.

### 2.4. Statistical Analysis

All statistical tests were performed using SPSS v.26.0 (SPSS Inc., Chicago, IL, USA). Quantitative variables are presented as the mean ± standard deviation, and qualitative data are presented as frequency (percentage). Descriptive statistics were used to characterize the sample. Between-group differences were determined using a Chi-square test for categorical variables. An ANOVA test was utilized to assess the associations between the categorical and numerical variables (assuming they were normally distributed, such as the levels of performance and age) after confirming the normality using the Kolmogorov Smirnov test. A simple linear regression test was used to measure the B coefficient for two continuous variables (such as age and score). The power of analysis (as determined through an ANOVA test) was utilized to measure the sample size, with the aim of detecting a difference of 1 in the mean score with a power of 80%, assuming a standard deviation of 0.5. A minimal sample size of 54 participants (18 for each group) was required. The statistical significance was set at *p* < 0.05.

## 3. Results

### 3.1. The General Demographic and Characteristics of the Participants

A total of 137 participants were included in this study. They possessed a mean age of 31.8 ± 0.5 years. The majority were women (62.8% vs. 37.2% men). Of the participants, 36.5% were single, while 63.5% were married. The mean number of years of experience was 6.9 ± 0.5 years. Our cohort comprised anesthesia physicians (30.0%), pediatric physicians (25.5%), and pediatric nurses (44.5%). Among the physicians, 27.6% were specialists/consultants and 72.4% were residents. Among the nurses, 78.7% held a bachelor’s degree and 21.3% had a master’s degree. Furthermore, nurses were identified in terms of workplace distribution, 57.4% of nurses worked in the intensive care unit (ICU), 14.8% in hospital wards, and 27.9% in the emergency department. Only 39.4% of the participants had attended pediatric pain management courses. Most participants used pain assessment tools in the hospital (73.3%). On average, participants worked 57.5 ± 2.1 h per week. Table 1 summarizes the general demographic and characteristics of the participants.

### 3.2. The Participants’ Responses to the Modified KASRP Questionnaire Assessing Knowledge and Attitudes Regarding Pediatric Pain Management

In the true/false section, most participants correctly identified common misconceptions in pain management, with 53–66% disagreeing with statements such as NSAIDs being ineffective for bone metastases, children under 11 being unable to self-report pain, opioid-naïve patients remaining on morphine for months postoperatively, gabapentin providing immediate relief after a single dose, opioid rotation requiring no dose adjustments, the lack of a role for simple analgesics alongside opioids, and the use of fentanyl patches as a first-line treatment for acute severe pain. Additionally, 68–92% correctly agreed with evidence-based statements, including those regarding the rarity of respiratory depression in patients on stable opioid doses, the benefits of multimodal analgesia, the potential influence of spiritual beliefs on pain perception, the need for individualized opioid titration, the limited role of benzodiazepines, the concept of equianalgesia, the importance of sedation monitoring during opioid therapy, the need to reassess patients with escalating analgesic requirements, and the delayed onset of the effect of neuropathic pain medications. However, misconceptions were evident, as more than half of the participants incorrectly agreed with statements such as vital signs being reliable indicators of pain intensity, young children having decreased pain sensitivity and limited memory of painful experiences, distraction implying the absence of severe pain, intravenous morphine providing 4–5 h of analgesia, patients needing to endure as much pain as possible before receiving opioids, the use of placebo injections to test for “real” pain, and withholding opioids during diagnostic evaluation to avoid masking the underlying cause. Additionally, the majority incorrectly disagreed with the correct statement that patients may sleep despite experiencing severe pain. Among the multiple-choice questions, the majority of participants correctly answered 9 out of 10 items. Between 50.4% and 86.2% selected the correct responses regarding the need to gradually wean postoperative patients off oral morphine before discharge, the use of IV opioids for brief episodes of severe pain (e.g., trauma or postoperative pain), the oral-to-IV morphine equivalence (30 mg oral ≈ 10 mg IV), the importance of administering postoperative analgesics on a fixed schedule, the likelihood that requests for additional analgesia reflect increased pain, the patient being the most accurate judge of their own pain intensity, the time-to-peak effect for IV (15 min) and oral morphine (1–2 h), and obstructive sleep apnea as a significant risk factor for opioid-induced respiratory depression. In contrast, only a minority correctly identified the signs of physical dependence following opioid discontinuation (sweating, yawning, diarrhea, and agitation). Participants scored an average of 20.7 ± 0.3 out of 34 (equivalent to 60.8 ± 1.1%). Performance was categorized as poor, fair, or good, with 22.6%, 64.2%, and 13.2% of participants falling into each category, respectively.

Table 2 presents the actual answers of the participants regarding the modified KASRP questionnaire and the correct answers regarding the modified KASRP questionnaire and the total score, respectively.

In terms of participants’ characteristics and whether they affect performance, professional role, level of physician responsibility, prior training in pediatric pain management, and the use of pain assessment tools yielded significant results. The mean final score was higher for anesthesia physicians (67.4 ± 1.9) compared to both pediatricians (60.8 ± 1.8) and pediatric nurses (56.2 ± 1.7), at *p* = 0.0001, and they were more frequently classified as good performers (*p* = 0.009). Similarly, specialists and consultants outperformed training residents, with higher mean scores (71.1 ± 2.4 vs. 61.8 ± 1.5, *p* = 0.002) and a greater proportion achieving good performance (*p* = 0.024). Both completion of pediatric pain management courses and the use of pain assessment tools were linked to higher performance, with *p*-values of *p* = 0.02 and *p* = 0.005, respectively. In contrast, demographic factors (age, gender, and marital status), professional experience, qualifications, workplace location, and working hours were not significantly associated with performance outcomes. Table 3 summarizes factors affecting the level of performance in terms of final percentage (out of 100%) and classifications of performance.

## 4. Discussion

To the best of our knowledge, this is the first study to evaluate this parameter among both physicians and nurses. Our study revealed an average score of 20.7 out of 34 (equivalent to 60.8) on the modified KASPR questionnaire, with most participants categorized as performing fairly on the questionnaire. More than half of the participants achieved a fair performance. Anesthesiologists scored higher than other specialties and were more frequently classified as good performers. In addition, specialists and consultants outperformed training residents, with higher mean scores and a greater proportion achieving good performance. Participants who attended pediatric pain management courses and those who reported using pain assessment tools performed better, although no significant difference was noticed in their average score.

The majority of our participants were categorized as having a fair performance, which aligns with findings from several recent studies reporting suboptimal knowledge and attitudes regarding pain management among healthcare providers. Similar knowledge gaps and negative attitudes regarding pain assessment and management have also been documented in studies from Jordan and other countries.

In Jordan, a cross-sectional study surveyed nursing students from different universities using the Pediatric Nurses’ Knowledge and Attitudes Survey (PNKAS) and reported poor performance, with a mean score of only 18.36%. This highlights the need for incorporating educational material regarding pain assessment and analgesia into nursing curricula [20]. Similarly, Abdel Razeq et al. discussed multiple obstacles hindering optimal pain management in pediatric patients with sickle cell disease, with workload, lack of psychological support, and limited time ranked among the most significant. These findings underscore the importance of addressing such barriers and their impact on patient care [21]. However, it was evident that medical care professionals who received pain education courses consistently achieved higher scores [11,21,22,23,24]. Collectively, these findings emphasize the need to integrate pain management into both undergraduate curricula and continuing professional training.

In concordance with this, international research using the KASRP questionnaire has similarly revealed poor levels of knowledge and attitudes toward pain management among healthcare providers [22,23,24,25,26,27,28]. Wang, Jiadong et al. reported an average score of 44.5% among Chinese nurses across 32 tertiary hospitals, which is notably lower than the performance score observed in our study [24]. In a study conducted in Sri Lanka in a national cancer institute, the majority of participants were categorized as having a poor level of knowledge (66.5%), with only 2.4% having a good level of knowledge, which suggests lower performance compared to our participants [27]. On the contrary, Alanizi, Amal Wanis et al. reported an average score of 25.62 out of 36 (71.16%), with findings comparable to ours [28].

Our findings mirror those of prior studies when it comes to specific misconceptions. For instance, misconceptions about the reliability of vital signs such as pain indicators, children’s pain sensitivity, and the role of placebo injections remain prevalent across multiple countries [24,27,28].

Notably, on the multiple-choice section, our participants performed relatively well compared to those in recent studies [24,27], with correct responses ranging from 50.4% to 86.2% in key clinical scenarios, specifically, in opioid pharmacology and postoperative pain management. However, consistent with earlier reports, recognition of physical dependence symptoms following opioid withdrawal remained poor in our sample, which is aligned with reports from studies from both Asia and the Middle East [27,28].

Clinician awareness is widely recognized as the most important factor in effectively mitigating pediatric pain. Multiple studies have highlighted this issue, with several identifying knowledge gaps in areas such as pain assessment, management, non-pharmacological interventions, and the use of analgesics. Reported barriers include a lack of appropriate assessment tools, underreporting of pain by parents, insufficient education and training on pediatric pain management, hesitancy and fear surrounding opioid use in pediatric populations, and the absence of interdisciplinary teams to provide pain relief and emotional support for families of seriously ill children [29,30,31].

Recent data suggest that while physicians are aware of the gap between optimal pediatric pain management and current practices, they are less likely than nurses to administer analgesia to infants during common procedures (such as heel prick, venipuncture, lumbar puncture, intubation, long-line insertion, and chest drain insertion). Although nurses report more frequent use of analgesia, nearly half do not administer it, indicating that procedural pain remains frequently underaddressed by healthcare professionals [32]. Additionally, limited availability of pain teams, coupled with institutional and legal constraints, negatively influenced the delivery of adequate analgesia [10]. These challenges highlight the need for organizational support and policy development to guarantee consistent and effective pain management practices in pediatric care settings.

Anesthesiologists have currently expanded their practice, which was formerly restricted to the operating room, to include perioperative medicine. The latter encompasses treatment of acute pain and postoperative and intensive care, in addition to chronic pain medicine, sleep medicine, and palliative care. Anesthesiology began to manage pain as a continuum, not being limited to the intraoperative period, and pain management is incorporated into their teaching curriculum [33,34,35]. This could explain the high scores of anesthesiologists in this study.

The main strength of this study is the inclusion of all healthcare providers managing pediatrics, including pediatricians, anesthesiologists, and pediatric nurses. Moreover, in comparison to other studies, the sample size is relatively adequate. Furthermore, the inclusion of several factors augments the results of the study. This study is not without limitations. Cross-sectional studies are have a lower quality of scientific evidence and results. Moreover, the relative exposure to pediatric patients is different among the included participants.

## 5. Conclusions

In conclusion, pediatric nurses (from all departments) have relatively less knowledge about acute pediatric pain management than physicians. In addition, anesthesiologists had the highest score, probably due to the pain management materials they studied during their training. Furthermore, attending pain management courses enhanced the levels of knowledge. Adequate interventions in healthcare institutions are essential, and healthcare schools should provide all healthcare providers, especially nurses, with adequate, updated, and accurate knowledge about the assessment and management of pain. Further research and healthcare policies should be encouraged to enhance the levels of pediatric pain management.

## Figures and Tables

**Table 1:** General demographic and characteristics of the participants.

Variables	Number (n) *	Percentage (%)
	Mean ± SEM	
**Age (years)**	31.8 ± 0.5	
**Gender**		
Male	51	37.2
Female	86	62.8
**Marital status**		
Single	50	36.5
Married	87	63.5
**Total experience (years)**	6.9 ± 0.5	
**Role**		
Anesthesia physician	41	30.0
Pediatric physician	35	25.5
Pediatric nurse	61	44.5
**Responsibility among physicians (out of 76)**		
Specialist/consultants	21	27.6
Training residents	55	72.4
**Qualification of nurses (out of 61)**		
Bachelor’s degree	48	78.7
Master’s degree	13	21.3
**Location of working for nurses (out of 61)**		
Intensive care unit	35	57.4
Ward	9	14.8
Emergency department	17	27.9
**Has received pediatric pain management courses**	54	39.4
**Uses pain assessment tools in the hospital**	101	73.7
**Average working hours per week (hours)**	57.5 ± 2.1	

* Number = 137.

**Table 2:** Actual answers of the participants and correct answers regarding the modified KASRP questionnaire and total scores.

Question	Response	Number	Percentage (%)
True/false questions			
Vital signs are always reliable indicators of the intensity of a patient’s pain.	True	88	64.2
	**False**	49	35.8
Because their nervous system is underdeveloped, children under two years of age have decreased pain sensitivity and limited memory of painful experiences.	True	76	55.5
	**False**	61	44.5
Patients who can be distracted from pain usually do not have severe pain.	True	84	61.3
	**False**	53	38.7
Patients may sleep in spite of severe pain.	**True**	55	40.1
	False	82	59.9
Aspirin and other nonsteroidal anti-inflammatory agents are NOT effective analgesics for painful bone metastases.	True	65	47.4
	**False**	72	52.6
Respiratory depression rarely occurs in patients who have been receiving stable doses of opioids over a period of months.	**True**	93	67.9
	False	44	32.1
Combining analgesics that work by different mechanisms (e.g., combining an NSAID with an opioid) may result in better pain control with fewer side effects than using a single analgesic agent.	**True**	123	89.8
	False	14	10.2
The usual duration of analgesia of 1–2 mg morphine IV is 4–5 h.	True	104	75.9
	**False**	33	24.1
Patients should be encouraged to endure as much pain as possible before using an opioid.	True	72	52.6
	**False**	65	47.4
Children less than 11 years old cannot reliably report pain so clinicians should rely solely on the parent’s assessment of the child’s pain intensity.	True	54	39.4
	**False**	83	60.6
Patients’ spiritual beliefs may lead them to think pain and suffering are necessary.	**True**	111	81.0
	False	26	19.0
After an initial dose of opioid analgesic is given, subsequent doses should be adjusted in accordance with the individual patient’s response.	**True**	122	89.1
	False	15	10.9
Giving patients sterile water by injection (placebo) is a useful test to determine if the pain is real.	True	83	60.6
	**False**	54	39.4
Post operatively a previously opioid naïve patient may stay on morphine at home for up to 3 months	True	48	35.0
	**False**	89	65.0
If the source of the patient’s pain is unknown, opioids should not be used during the pain evaluation period, as this could mask the ability to correctly diagnose the cause of pain.	True	100	73.0
	**False**	37	27.0
Anticonvulsant drugs such as gabapentin (Neurontin) produce optimal pain relief after a single dose.	True	46	33.6
	**False**	91	66.4
Benzodiazepines are not effective pain relievers and are rarely recommended as part of an analgesic regiment.	**True**	95	69.3
	False	42	30.7
The term ‘equianalgesia’ means approximately equal analgesia and is used when referring to the doses of various analgesics that provide approximately the same amount of pain relief.	**True**	109	79.6
	False	28	20.4
Sedation assessment is recommended during opioid pain management because excessive sedation precedes opioid-induced respiratory depression.	**True**	125	91.2
	False	12	8.8
In the hospital if there is a sudden escalation in analgesic requirements, it warrants re-evaluating the patient for possible new causes/pathologies.	**True**	126	92.0
	False	11	8.0
Medications for neuropathic pain such as gabapentin, pregabalin and TCAs might need days to reach peak effect	**True**	109	79.6
	False	28	20.4
When rotating from one opioid to another we use equianalgesic doses without changes in those doses.	True	60	43.8
	**False**	77	56.2
If you use opioids there is no role for simple analgesics such as paracetamol or NSAIDs	True	48	35.0
	**False**	89	65.0
Fentanyl patches can be used as a first line choice in treating severe acute pain	True	51	37.2
	**False**	86	62.8
**Multiple-choice questions**			
For a patient who is for discharge after an operation, but is currently on oral morphine for his post operative pain, what is a safe recommendation regarding analgesia	a. Cease morphine on discharge and continue on simple analgesics.	39	28.5
	b. After discharge it is a safe assumption that the patient will cease morphine on his own without specific guidance.	9	6.5
	**c. Wean off morphine gradually**	89	65.0
The recommended route administration of opioid analgesics for patients with brief, severe pain of sudden onset such as trauma or postoperative pain is	**a. intravenous**	118	86.2
	b. intramuscular	7	5.1
	c. subcutaneous	5	3.6
	d. oral	7	5.1
A 30 mg dose of oral morphine is approximately equivalent to:	a. Morphine 5 mg IV	53	38.7
	**b. Morphine 10 mg IV**	69	5.4
	c. Morphine 30 mg IV	15	10.9
	d. Morphine 60 mg IV	0	0.0
Analgesics for post-operative pain should initially be given	**a. around the clock on a fixed schedule**	87	63.5
	b. only when the patient asks for the medication	32	23.4
	c. only when the nurse determines that the patient has moderate or greater discomfort	18	13.1
The most likely reason a patient with pain would request increased doses of pain medication is	**a. The patient is experiencing increased pain.**	78	56.9
	b. The patient is experiencing increased anxiety or depression.	21	15.4
	c. The patient is requesting more staff attention.	8	5.8
	d. The patient’s requests are related to addiction.	30	21.9
The most accurate judge of the intensity of the patient’s pain is	a. the treating physician	24	17.5
	b. the patient’s primary nurse	19	13.9
	**c. the patient**	86	62.8
	d. the pharmacist	3	2.2
	e. the patient’s spouse or family	5	3.6
The time to peak effect for morphine given IV is	**a. 15 min.**	104	75.9
	b. 45 min.	25	18.3
	c. 1 h	7	5.1
	d. 2 h	1	0.7
The time to peak effect for morphine given orally is	a. 5 min.	7	5.1
	b. 30 min.	37	27.0
	**c. 1–2 h**	89	65.0
	d. 3 h	4	2.9
Following abrupt discontinuation of an opioid, physical dependence is manifested by the following:	**a. sweating, yawning, diarrhea and agitation with patients when the opioid is abruptly discontinued.**	32	23.4
	b. Impaired control over drug use, compulsive use, and craving.	13	9.5
	c. The need for higher doses to achieve the same effect.	12	8.8
	d. a and b	80	58.4
Which statement is true regarding opioid induced respiratory depression:	a. More common several nights after surgery due to accumulation of opioid.	21	15.3
	**b. Obstructive sleep apnea is an important risk factor.**	71	51.8
	c. Occurs more frequently in those already on higher doses of opioids before surgery.	32	23.4
	d. Can be easily assessed using intermittent pulse oximetry.	13	9.5
**Total scores and classifications**			
**Mean average of participants performance (score and percentage) ± SEM**		**Score (out of 34)**	**Percentage (100)** **(100 100%)**
		20.7 ± 0.3	60.8 ± 1.1
**Participants classification according to performance**	**Poor**	31	22.6
	**Fair**	88	64.2
	**Good**	18	13.2

The correct answers are underlined and bold.

**Table 3:** Factors affecting the level of performance in terms of final percentage (out of 100%) and classifications of performance.

Variables	Mean ± SEM * or B Regression Coefficient ± SEM ** or Number (Percentage) ***					
	Final Score (Percentage)	*p*-Value	Poor Performance (n = 31)	Fair Performance (n = 88)	Good Performance (n = 18)	*p*-Value
**Age (years)**	0.083 ± 0.2	0.67 **	31.7 ± 0.9	31.6 ± 0.6	32.8 ± 1.1	0.74 *
**Gender**						
Male	63.6 ± 1.8	0.054 *	9 (17.6)	34 (66.7)	8 (15.7)	0.51 ***
Female	59.1 ± 1.4		22 (25.6)	54 (62.8)	10 (11.6)	
**Marital status**						
Single	61.5 ± 1.7	0.63 *	8 (16.0)	37 (74.0)	5 (10.0)	0.19 ***
Married	60.3 ± 1.5		23 (26.4)	51 (58.6)	13 (14.9)	
**Total period of experience (years)**	−0.15 ± 0.1	0.45 **	7.5 ± 0.9	6.6 ± 1.2	7.0 ± 0.6	0.78 *
**Role**						
Anesthesia physician	67.4 ± 1.9	0.0001 *	3 (7.3)	31 (75.6)	7 (17.1)	0.009 ***
Pediatric physician	60.8 ± 1.8		6 (17.1)	25 (71.4)	4 (11.4)	
Pediatric nurse	56.2 ± 1.7		22 (36.1)	32 (52.5)	7 (11.5)	
**Responsibility among physicians**						
Specialist/consultants	71.1 ± 2.4	0.002 *	0 (0.0)	15 (71.4)	6 (28.6)	0.024 ***
Training residents	61.8 ± 1.5		9 (16.4)	41 (74.5)	5 (91.)	
**Qualification of nurses**						
Bachelor’s degree	56.0 ± 1.9	0.81 *	19 (39.6)	22 (45.8)	7 (14.6)	0.10 ***
Master’s degree	57.1 ± 2.1		3 (23.1)	10 (76.9)	0 (0.0)	
**Location of working for nurses**						
Intensive care unit	56.9 ± 2.2	0.39 *	11 (31.4)	20 (57.1)	4 (11.4)	0.64 ***
Ward	59.8 ± 3.1		3 (33.3)	4 (44.4)	2 (22.2)	
Emergency department	52.8 ± 2.3		8 (47.1)	8 (47.1)	1 (5.9)	
**Has received pediatric pain management courses**	60.7 ± 2.0	0.95 *	17 (31.5)	27 (50.0)	10 (18.5)	0.02 ***
**Uses pain assessment tools in the hospital**	60.3 ± 1.4	0.48 *	27 (26.7)	57 (56.4)	17 (16.8)	0.005 ***
**Average working hours per week (hours)**	0.04 ± 0.04	0.40 **	50.6 ± 2.6	60.4 ± 2.9	55.2 ± 2.1	0.14 *

* ANOVA test. ** Linear regression test. *** Chi-square test. Significant if *p-*value < 0.05.

## Data Availability

The datasets used and/or analyzed during the current study are presented in the tables of this study.

## Supplementary Materials

The following supporting information can be downloaded at: https://www.mdpi.com/article/10.3390/healthcare13202570/s1. Table S1. Knowledge and Attitudes Survey Regarding Pain.

## Abbreviations

KASRP: Knowledge and Attitude Survey Regarding Pain; ICU intensive care unit; IASP; International Association for the Study of Pain; MCQs: multiple-choice questions
