# Supplementary material for: Assessment of Knowledge and Attitudes Regarding Acute Pediatric Pain Management Among Anesthesiologists, Pediatricians, and Pediatric Nurses: A Cross-Sectional Study from Jordan
Source: Healthcare (Basel). 2025 Oct 13;13(20):2570. doi: 10.3390/healthcare13202570 (PMC12562588; doi:10.3390/healthcare13202570)
Supplement: Supplementary file 1 [file healthcare-13-02570-s001.zip › healthcare-3887715-supplementary.pdf]

Table S1. Knowledge and Attitudes Survey Regarding Pain

Question	Response	Correct response	Original/Added
**True/false questions**			
Vital signs are always reliable indicators of the intensity of a patient's pain.	True<br>False	False	Original
Because their nervous system is underdeveloped, children under two years of age have decreased pain sensitivity and limited memory of painful experiences.	True<br>False	False	Original
Patients who can be distracted from pain usually do not hav e severe pain.	True<br>False	False	Original
Patients may sleep in spite of severe pain.	True<br>False	True	Original
Aspirin and other nonsteroidal anti-inflammatory agents are NOT effective analgesics for painful bone metastases.	True<br>False	False	Original

Respiratory depression rarely occurs in patients who have been receiving stable doses of opioids over a period of months.	True<br>False	True	Original
Combining analgesics that work by different mechanisms (e.g., combining an NSAID with an opioid) may result in better pain control with fewer side effects than using a single analgesic agent.	True<br>False	True	Original
The usual duration of analgesia of 1-2 mg morphine IV is 4-5 hours.	True<br>False	False	Original
Patients should be encouraged to endure as much pain as p ossible before using an opioid.	True<br>False	False	Original
Children less than 11 years old cannot reliably report pain so clinicians should rely solely on the parent's assessment of the child's pain intensity.	True<br>False	False	Original
Patients' spiritual beliefs may lead them to think pain and suffering are necessary.	True<br>False	True	Original
After an initial dose of opioid analgesic is given, subsequent doses should be adjusted in accordance with the individual patient's response.	True<br>False	True	Original

Giving patients sterile water by injection (placebo) is a useful test to determine if the pain is real.	True<br>False	False	Original
Post operatively a previously opioid naïve patient may stay on morphine at home for up to 3 months	True<br>False	False	**Added**
If the source of the patient's pain is unknown, opioids should not be used during the pain evaluation period, as this could mask the ability to correctly diagnose the cause of pain.	True<br>False	False	Original
Anticonvulsant drugs such as gabapentin (Neurontin) produce optimal pain relief after a single dose.	True<br>False	False	Original
Benzodiazepines are not effective pain relievers and are rarely recommended as part of an analgesic regiment.	True<br>False	True	Original
The term 'equianalgesia' means approximately equal analge sia and is used when referring to the doses of various analgesics that provide approximately the same amount of pain relief.	True<br>False	True	Original
Sedation assessment is recommended during opioid pain	True		

management because excessive sedation precedes opioid-induced respiratory depression.	False	True	Original
In the hospital if there is a sudden escalation in analgesic requirements, it warrants re-evaluating the patient for possible new causes/ pathologies.	True<br>False	True	**Added**
Medications for neuropathic pain such as gabapentin, pregabalin and TCAs might need days to reach peak effect	True<br>False	True	**Added**
When rotating from one opioid to another we use equianalgesic doses without changes in those doses.	True<br>False	False	**Added**
If you use opioids there is no role for simple analgesics such as paracetamol or NSAIDs	True<br>False	False	**Added**
Fentanyl patches can be used as a first line choice in treating severe acute pain	True<br>False	False	**Added**

Multiple choice questions			
For a patient who is for discharge after an operation, but is currently on oral morphine for his post operative pain, what is a safe recommendation regarding analgesia	a. Cease morphine on discharge and continue on simple analgesics.<br><br>b. After discharge it is a safe assumption that the patient will cease morphine on his own without specific guidance.<br><br>c. Wean off morphine gradually	c. Wean off morphine gradually	**Added**
The recommended route administration of opioid analgesics for patients with brief, severe pain of sudden onset such as trauma or postoperative pain is	a. intravenous<br><br>b. intramuscular<br><br>c. subcutaneous<br><br>d. oral	a. intravenous	Original
A 30 mg dose of oral morphine is approximately equivalent to:	a. Morphine 5 mg IV<br><br>b. Morphine 10 mg IV<br><br>c. Morphine 30 mg IV<br><br>d. Morphine 60 mg IV	b. Morphine 10 mg IV	Original

Analgesics for post-operative pain should initially be given	a. around the clock on a fixed schedule<br><br>b. only when the patient asks for the medication<br><br>c. only when the nurse determines that the patient has moderate or greater discomfort	a. around the clock on a fixed schedule	**Added**
The most likely reason a patient with pain would request increased doses of pain medication is	a. The patient is experiencing increased pain.<br><br>b. The patient is experiencing increased anxiety or depression.<br><br>c. The patient is requesting more staff attention.<br><br>d. The patient's requests are related to addiction.	a. The patient is experiencing increased pain.	Original
The most accurate judge of the intensity of the patient's pain is	a. the treating physician<br><br>b. the patient's primary nurse<br><br>c. the patient	c. the patient	Original

	d. the pharmacist<br><br>e. the patient's spouse or family		
The time to peak effect for morphine given IV is	a. 15 min.<br><br>b. 45 min.<br><br>c. 1 hour<br><br>d. 2 hours	a. 15 min.	Original
The time to peak effect for morphine given orally is	a. 5 min.<br><br>b. 30 min.<br><br>c. 1 – 2 hours<br><br>d. 3 hours	c. 1 – 2 hours	Original
Following abrupt discontinuation of an opioid, physical dependence is manifested by the following:	a. sweating, yawning, diarrhea and agitation with patients when the opioid is abruptly discontinued.<br><br>b. Impaired control over drug use, compulsive use, and craving.	a. sweating, yawning, diarrhea and agitation with patients when the opioid is abruptly discontinued.	Original

	c. The need for higher doses to achieve the same effect.<br><br>d. a and b		
Which statement is true regarding opioid induced respiratory depression:	a. More common several nights after surgery due to accumulation of opioid.<br><br>b. Obstructive sleep apnea is an important risk factor.<br><br>c. Occurs more frequently in those already on higher doses of opioids before surgery.<br><br>d. Can be easily assessed using intermittent pulse oximetry.	b. Obstructive sleep apnea is an important risk factor.	Original
